# The Pathology and Treatment of Epilepsy

**Published:** 1887-04

**Authors:** 


### THE PATHOLOGY AND TREATMENT OF EPILEPSY.

At a recent meeting of the New York Academy of Medicine, Professor Wm. H. Thomson, of the University Medical School, read a paper on the "Pathology and Treatment of Epilepsy," based on notes of sixty consecutive cases in practice, in which he advanced some rather unusual views, among which was the opinion that all convulsive seizures of an epileptiform character, whether due to a temporary peripheral irritation or not, as convulsions from dentition, for example, really belong to true epilepsy.

Dr. Thomson regards *suddenness* as the invariable and essential element in epilepsy; it is the single truly sudden disease, the only affections resembling it in this particular being laryngismus stridulus and spasmodic asthma, though in these the suddenness is found not to be absolute as in epilepsy. Apoplexy, hemiplegia, sunstroke, etc., being accidents, cannot be strictly compared with epilepsy; nor are hysterical and neuralgic attacks so sudden as those of epilepsy. Furthermore, Dr. Thomson regards *petit mal* as the most real form of the disease. In regard to the cell discharge or explosion theory, the views of Hughlings-Jackson, and Nothnagel's "convulsive centre," he thinks that if no other form of epilepsy than *petit mal* had ever been observed, the explosion theory would never have been proposed. With Jackson and Gowers he is willing to grant that there is a motor discharge in every convulsive seizure; but that it is a different matter to say that an attack of epilepsy is a motor discharge. He believes all motor phenomena except the voluntary to be under the control of sensory impulse, and a sudden suspension of the regulating sensory impression may result from a variety of causes. Any irregular motor phenomena are, therefore, due to a loss of the customary sensory influence; and this, he thinks, explains the clinical facts of epilepsy without the necessity of supposing any additional nervous force being called into action.

He considers the phenomena of epilepsy to be the effect of an afferent sensory impression when some abnormal condition of the nerve centres is present. What this condition may be he is not prepared to say, but it seems most probable to him that it is one of malnutrition. If asked if he would assert that all cases of epilepsy are attended with sensory impressions in the face of the well-known fact that in certain instances there are definite lesions of the brain present, he would reply that we do not get rid of the sensory element when we enter the cranial cavity. A syphilitic gumma of the brain may be as truly an excitant of sensory irritability as an external impression. He does not hesitate to acknowledge that a motor centre may be excited by the application of an electric current after trephining the skull; but the explanation of the phenomena noted he believes to be found in the fact of a wholly unaccustomed irritation in a centre habituated to act in response to sensory impressions. The hypothesis of a sudden suspension of the ordinary suspensory functions, he thinks, fully accounts for all the phenomena observed in epilepsy.

As regards his treatment of epilepsy, Dr. Thomson says that of late years, since he has based his therapeutic measures on the hypothesis that the lesion of epilepsy is to be found in the sensory, rather than the motor, centres, he has grown much less skeptical of the advantages of treatment in this disease than formerly. The first thing to be aimed at is the improvement of nerve-nutrition; and by far the best agent for this purpose is cod-liver oil, which he prescribes as regularly in epilepsy as in phthisis. Phosphorus is also a useful remedy in this connection. Like the great mass of physicians, he has found the bromides the best agents for controlling peripheric irritation, and he has found cod-liver oil of very good service in counteracting their debilitating effects. When there is persistent cortical irritation, as indicated by muscular twitchings during sleep, he employs, with happy results, the bichloride of mercury, or the oleate by inunction. He uses belladonna or oxide of zinc in all cases in which the attacks show any connection with disturbances in the alimentary canal, and when there is reflex irritability, he uses chloral hydrate or Hoffman's anodyne, in addition to the bromides. Digitalis he uses in all cases characterized by vascular disturbance, or where there is involuntary discharge of urine during the attack. He is also in the habit of using, in a certain proportion of cases, a red pepper pack at night; one drachm of capsicum being used to the pint of hot water for this purpose. In one case, a patient who ordinarily had two epileptic attacks a day did not have a single one for seven weeks after this measure was resorted to; the disease being completely arrested by the peripheral excitation thus secured. In his opinion, an important part of the treatment is the total exclusion of all butcher meat for a period of two years; though poultry and fish are permissible. Animal diet, he believes, predisposes to convulsions in direct proportion to the quantity in which it is used. The tendency to convulsions in the carnivoræ, and the absence of this in herbivorous animals, are apparently due to the respective diet in each class. Another thing to be avoided is eating fast, as the too rapid mastication and swallowing of food seems to act directly on the convulsive centre of the medulla oblongata. It is possible that the habit of eating too fast may thus induce confirmed epilepsy. These points show, he thinks, the direction in which efforts should be made by which better results may be expected in the future, and the treatment thus be relieved of the grievous burden of suspicion which it has borne so long.

It could scarcely be supposed that such views as to the pathology of epilepsy could be expressed before the Academy without discussion. It may be questioned, as Dr. Putzel remarked, whether anything can be considered epilepsy unless the convulsive habit is established. But we cannot so certainly draw a line of demarkation between infantile convulsions and true epilepsy, as Dr. Putzel seems to think, as clinical experience shows that the tendency to convulsions in children is closely associated with the epileptic diathesis, and that accidental convulsions are often the beginning of lifelong epilepsy; and we also know that many cases of epilepsy give a history of convulsions in childhood. But a consideration of the pathology of epilepsy, and epileptiform convulsions, would carry us beyond the limits of this article.

With one exception, it seems that Dr. Thomson's views as to the treatment of epilepsy were generally endorsed. Dr. Wm. H. Draper, however, did not agree as to the danger of animal food; on the contrary, he is inclined to think that the origination of an attack is more likely to follow the ingestion of starchy foods, and he prefers that his patients should use animal food and milk, and a diminished quantity of the carbo-hydrates; and from this he has had very good results.

At the same meeting of the Academy, Dr. A. D. Rockwell read a paper on "The Value of Electricity in the Treatment of Epilepsy," in which, among others, he drew the following conclusions:

Electricity possesses a certain value in the treatment of epilepsy.

It is not claimed that it can alone cure the disease, but in many instances it is of great service as an adjuvant to the bromides.

In the nocturnal variety, its good effects are especially marked.

The methods of application to be used are central galvanization and general faradization.

It is important that the agent should be administered with great care. Anything like a shock should be avoided, and the applications should not be continued too long at a time.

The treatment should be kept up, with suitable intermissions, for two years after all epileptic symptoms have disappeared.—*Journal of American Med. Asso.*

